# Multidimensional Single Cell Based STAT Phosphorylation Profiling Identifies a Novel Biosignature for Evaluation of Systemic Lupus Erythematosus Activity

**DOI:** 10.1371/journal.pone.0021671

**Published:** 2011-07-22

**Authors:** Xinfang Huang, Yanzhi Guo, Chunde Bao, Nan Shen

**Affiliations:** Renji Hospital, Shanghai Jiao Tong University School of Medicine, Shanghai, China; Fundação Oswaldo Cruz, Brazil

## Abstract

**Introduction:**

Dysregulated cytokine action on immune cells plays an important role in the initiation and progress of systemic lupus erythematosus (SLE), a complex autoimmune disease. Comprehensively quantifying basal STATs phosphorylation and their signaling response to cytokines should help us to better understand the etiology of SLE.

**Methods:**

Phospho-specific flow cytometry was used to measure the basal STAT signaling activation in three immune cell types of peripheral-blood mononuclear cells from 20 lupus patients, 9 rheumatoid arthritis (RA) patients and 13 healthy donors (HDs). A panel of 27 cytokines, including inflammatory cytokines, was measured with Bio-Plex™ Human Cytokine Assays. Serum Prolactin levels were measured with an immunoradiometric assay. STAT signaling responses to inflammatory cytokines (interferon α [IFNα], IFNγ, interleukin 2 [IL2], IL6, and IL10) were also monitored.

**Results:**

We observed the basal activation of STAT3 in SLE T cells and monocytes, and the basal activation of STAT5 in SLE T cells and B cells. The SLE samples clustered into two main groups, which were associated with the SLE Disease Activity Index 2000, their erythrocyte sedimentation rate, and their hydroxychloroquine use. The phosphorylation of STAT5 in B cells was associated with cytokines IL2, granulocyte colony-stimulating factor (G-CSF), and IFNγ, whereas serum prolactin affected STAT5 activation in T cells. The responses of STAT1, STAT3, and STAT5 to IFNα were greatly reduced in SLE T cells, B cells, and monocytes, except for the STAT1 response to IFNα in monocytes. The response of STAT3 to IL6 was reduced in SLE T cells.

**Conclusions:**

The basal activation of STATs signaling and reduced response to cytokines may be helpful us to identify the activity and severity of SLE.

## Introduction

Systemic lupus erythematosus (SLE) is an autoimmune disease of unknown etiology. Dysregulated cytokine release plays an important role in the etiology of SLE and also correlates with its progression. Inflammatory cytokines, such as interleukin 6 (IL6,) IL10, interferon α (IFNα), and IL12, are elevated in the sera of SLE patients [1]–[3]. Jak-STAT [the activation of the Janus kinases (Jaks) and their downstream signal transducer and activator of transcription (Stat) proteins] signaling play a critical role in mediating the biological response to most of these cytokines.

When cytokines bind their specific receptors, the JAKs are activated by phosphorylating specific receptor tyrosine motifs. STATs are then activated by the JAK-dependent tyrosine phosphorylation. Activated STATs finally translocate to the nucleus, start the transcription of genes related [4]–[5].

The abnormal of STAT signaling pathway in immune cells of SLE has been reported before. T cells from patients with SLE display increased levels of total and phosphorylated STAT3, which is located primarily in the nucleus [6]. Elevated total STAT1 protein or its activated/phosphorylated form have also been detected in kidney samples from patients with diffuse proliferative lupus nephritis (DPLN) and MRL/lpr mice with lupus nephritis [7]–[8]. STAT-1 expression was also be found increased in peripheral-blood mononuclear cells (PBMC)from SLE patients and correlated significantly with disease activity. SLE monocytes showed a considerably higher increase in pSTAT-1 expression upon IFNγ stimulation than monocytes from healthy individuals [9].

However, little is known about the profiles of phosphorylated STATs (pSTATs) in the PBMC of SLE patients. Measuring pSTATs levels in immune cell subtypes should extend our understanding of the JAK–STAT signaling network in the etiology of SLE as well as in other disease such as cancer [10]–[11] and in human immunodeficiency virus (HIV) infection [12]. The technique of phospho-specific flow cytometry measures the phosphorylation state of intracellular proteins at the single cell level has been verified as a rapid and reliable tool for measuring intracellular signaling proteins at the single cell level [13]–[17].

Here we applied phospho-specific flow cytometry to measure the basal STATs phosphorylation and their signaling response in the major three immune cell types at the single cell in SLE patients.

## Materials and Methods

### Ethics Statement

Ethics approval for this study was obtained from the Ethics Committee of Shanghai Renji Hospital, Shanghai Jiaotong University School of Medicine.

### Subjects

We recruited 20 lupus patients, 9 RA (rheumatoid arthritis), 13 normal controls, after obtaining their informed consent. Participants with a current or recent infection were excluded from the study (Table 1). All SLE and RA patients fulfilled the American College of Rheumatology classification criteria for SLE [18] and RA [19]. The SLE Disease Activity Index 2000 (SLEDAI-2K) score for each patient was determined at the time of blood collection [20] (Table 1). The average prednisone dose taken by all the SLE patients was 17.38 mg/day, one patient had not been treated with immunosuppressants, four patients were not receiving steroid therapy, eight patients were receiving an antimalarial drug (hydrochloroquine 200–400 mg/day), and three patients were taking 5–7.5 mg of methotrexate per week. No patient was using another immunosuppressant, such as mycophenolate mofetil, cyclophosphamide, or cyclosporin A. None of the RA patients received steroid therapy. The patients were taking 7.5–15 mg of methotrexate per week. No patient was using biologic agents or lefluomide.

**Table 1:** Demographics of SLE and RA patients and healthy donors.

	SLE patients (n = 20)	RA (n = 9)	HDs (n = 13)
Age (years)	33.75±2.558	33.78±3.479	27.92±0.4995
Sex, female (%)	17 (85%)	1 (88.89%)	11 (84.62%)
Disease duration (years)	5.319±1.502	4.889±1.701	_
SLEDAI-2K	7.350±6.714	_	_

Except where otherwise indicated, the values expressed are means ± standard errors of the means (ranges). There were no significant differences between the patients with SLE, the patients with RA, and the healthy donors in terms of their age and sex. SLE, systemic lupus erythematosus; RA, rheumatoid arthritis; SLEDAI-2K, SLE Disease Activity Index 2000; HDs, healthy donors.

### Antibodies and flow cytometry

The following antibodies were used in this study, and were purchased from BD PharMingen (San Diego, CA): anti-pStat1(pY701), anti-pStat3(pY705), anti-pStat5(pY694), anti-CD3, anti-CD14, and anti-CD19. Lyse/Fix Buffer, Perm Buffer II, and Staining Buffer (FBS) were also purchased from BD PharMingen.

For the ex vivo stimulation experiments, fresh PBMC were cultured in complete RPMI 1640 medium (Gibco, Invitrogen, Carlsbad, CA, USA) containing 10% fetal calf serum (FCS), 100 U/mL penicillin (Gibco), and 100 µg/mL streptomycin (Gibco). The PBMC were stimulated for 15 min with 500 U/mL IFNα, 10 ng/mL IFNγ, 50 U/mL IL2, 200 ng/mL IL6, or 10 ng/mL IL10. The PBMC were then fixed in Lyse/Fix Buffer at 37°C for 15 min.

The staining experiments were performed according to the manufacturer's protocol. In brief, human PBMC, unstimulated or stimulated with the cytokines cited above, were fixed in Lyse/Fix Buffer (BD Biosciences) at 37°C for 15 min, and washed once with 2% FCS. The PBMC were permeabilized with cold Perm Buffer II for 30 min on ice, washed once with phosphate-buffered saline, then stained with primary antibody for 30 min, and washed twice with 2% FCS before analysis. The data were analyzed using CellQuest software (BD Biosciences, Mountain View, CA).

### Cytokine measurements

Blood samples were taken from the patients and healthy donors (HDs) after an overnight fast. Hemolyzed samples were discarded. We measured IL1β, IL1 receptor α (IL1rα), IL2, IL4, IL5, IL6, IL7, IL8, IL10, IL12(p70), IL13, IL15, IL17, basic fibroblast growth factor, eotaxin, granulocyte colony-stimulating factor (G-CSF), granulocyte–macrophage colony-stimulating factor (GM-CSF), interferon γ (IFNγ), monocyte chemotactic protein 1, macrophage inflammatory proteins 1α (MIP1α), MIP1β, human platelet-derived growth factor BB, RANTES (regulated upon activation of normal T cell expressed and secreted), tumor necrosis factor α (TNFα), and vascular endothelial growth factor using Bio-Plex™ Human Cytokine Assays (27-Plex panel; Bio-Rad, Hercules, CA). The samples were analyzed with the Bio-Plex Manager Software (Bio-Rad). Prolactin (PRL) levels were assessed with a chemiluminescent assay (Immulite/Immulite 1000 n, Siemens Medical Solutions Diagnostics). The cytokines were measured in a single well.

### Statistical analysis

The data were analyzed using the Prism4 software, version 4.03 (GraphPad, CA, USA). An Unpaired Student's t-tests were used, provided that the data followed Gaussian distributions. Mann-Whitney tests were used when the data were not normally distributed. Pearson and Spearman correlation coefficients were used for correlation studies between variables that were or were not normally distributed. P values less than 0.05 were considered significant. SLE samples were clustered using the HCE3.5 package (Hierarchical Cluster Explorer) [21].

## Results

### Activation of STAT signaling in SLE patients

**Figure 1:**
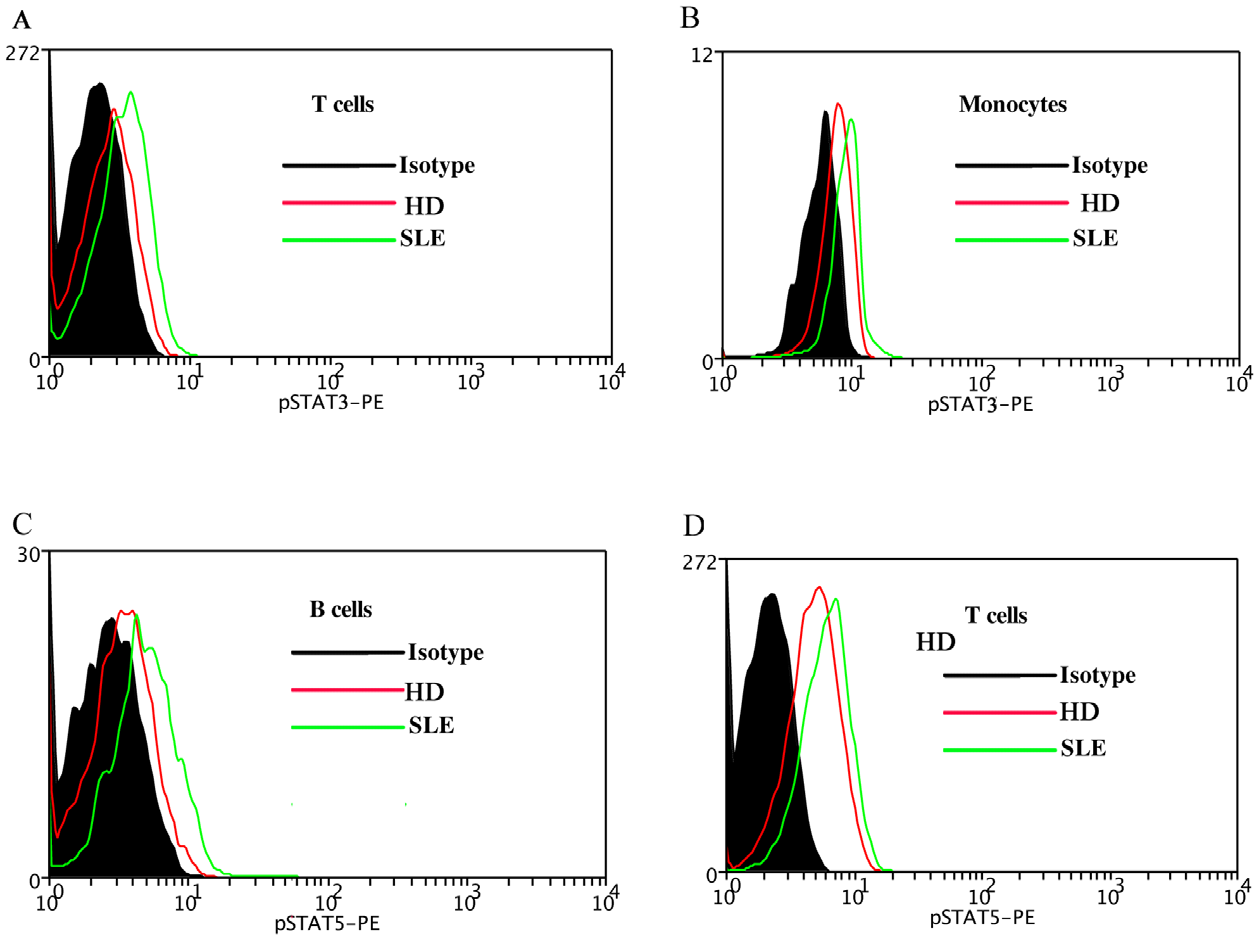
pSTATs expression in SLE patient and healthy donor (HD). A representative experiment is shown. The MFI of pSTAT3 in the T cells (1A), monocytes (1B) of SLE patient was compared with that of HD. The MFI of pSTAT5 in the B cells (1C), T cells (1D) of SLE patient was compared with that of HD. MFI, mean fluorescence index; SLE, systemic lupus erythematosus; HD, healthy donor.

Peripheral anticoagulated blood cells were lysed, fixed, and stained with antibodies specific for the surface markers CD3, CD14, and CD19. The phosphorylation of STAT1, STAT3, and STAT5 on the activating residues was measured and compared in three subsets of immune cells (CD3^+^ T cells, CD19^+^ B cells, and CD14^+^ monocytes) in 20 SLE patients, 9 RA patients and 13 HDs (Figure 1A). The SLE patients, RA patients and HDs did not differ significantly with respect to their mean ages or sex distributions (Table 1). The mean SLEDAI-2K score of 7.35 indicates that the SLE patients had moderate disease activity (Table 1). The median fluorescence intensity (MFI) for each pSTAT in the SLE, RA patients was calculated and compared with that of the HDs.

**Figure 2:**
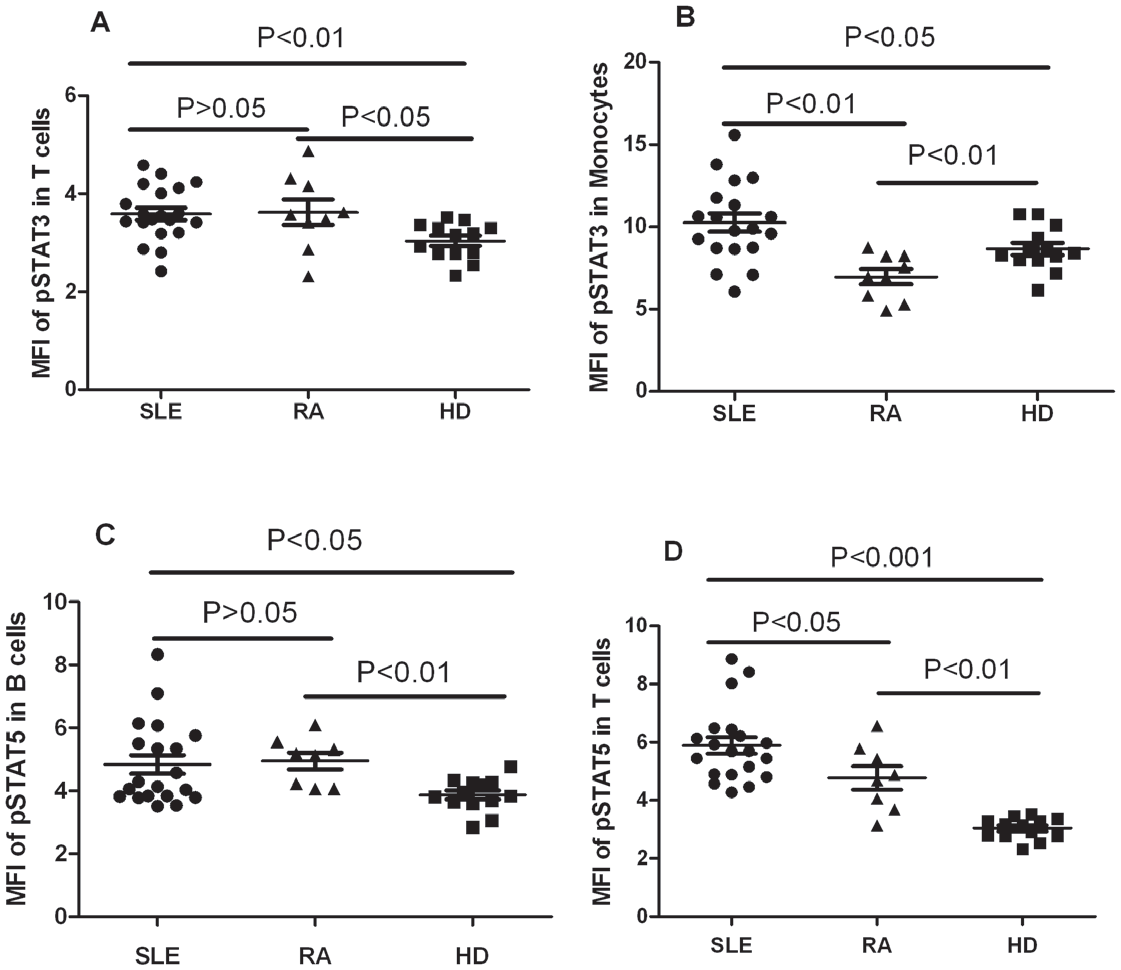
Comparison of pSTATs expression between SLE, RA patients and healthy donors (HDs). Each symbol represents an individual patient or HDs. (A) The MFI of pSTAT3 was elevated in the T cells of SLE, RA patients relative to that in HDs. (B) The MFI of pSTAT3 was elevated in the monocytes of SLE patients, but decreased in the monocytes of RA as compared with that in HDs. (C) The B cells of SLE patients, RA patients showed a higher MFI for pSTAT5 than that in HDs. (D) The T cells of SLE patients, RA patients showed a higher MFI for pSTAT5 than that in HDs. MFI, mean fluorescence index; SLE, systemic lupus erythematosus; RA, rheumatoid arthritis;HDs, healthy donors.

Increased activation of STAT3 was observed in SLE, RA T cells (SLE 3.584±0.1243, RA 3.620±0.2553, HD 3.034±0.1026, SLE vs HD, P<0.01, RA vs HD P<0.05, Figure 1A, Revised Figure 2A). SLE monocytes had significantly higher activation of STAT3 than disease control RA and healthy donors. The expression level of phosphorylation of STAT3 in Monocytes of RA showed lower than SLE and HD (SLE 10.25±0.5551, RA 6.946±0.4587, HD 8.648±0.3694, SLE vs HD P<0.05, SLE vs RA, P<0.01, RA vs HD, P<0.01, Figure 1B, Revised Figure 2B). Increased activation of STAT5 was observed in SLE, RA B cells and T cells. (B cells: SLE 4.837±0.2955, RA 4.945±0.2672, HD 3.866±0.1453, SLE vs HD P<0.05, RA vs HD P<0.01, Figure 1C, Revised 2C; T cells: SLE 5.889±0.2864, RA 4.774±0.4005, HD 3.034±0.1026, SLE vs HD, P<0.001, RA vs HD P<0.01, Figure 1D, Revised Figure 2D). The expression level of phosphorylation of STAT5 in T cells showed lower in RA than SLE (P<0.05, Revised Figure 2D). Our data indicated the activation of STAT3, STAT5 signaling in PBMC of SLE patients. There existed disease-specific profiling of PBMC pSTATs. There was activation of STAT3 in the monocytes of SLE patients, but this STAT3 signaling was inhibited in the monocytes of RA patients (Revised Figure 2B).

No STAT1 activation was detected in any of the three major immune-cell types.

### Activation of basal STAT signaling is associated with SLE activity

**Figure 3:**
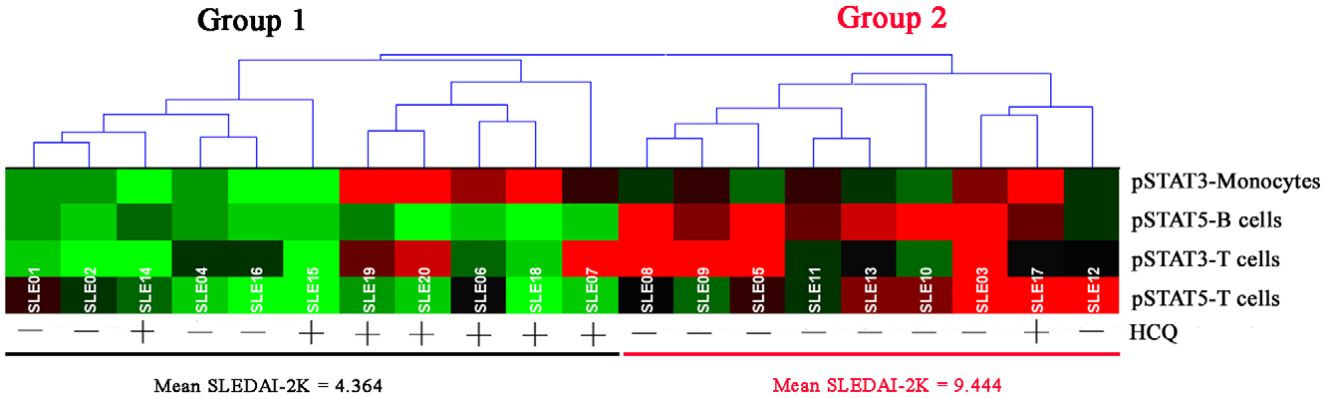
SLE samples were clustered by the expression of basal phosphorylated STAT3 and STAT5. Each grid represents the phosphorylation of a STAT protein in one cell type. The phosphorylation of each STAT protein was scaled relative to the minimum phosphorylation level among the 20 SLE samples.The 20 SLE patient samples were grouped according to their similarities using hierarchical clustering. Two groups of SLE patients were identified, based on similarities in the expression of basal phosphorylated STAT3 and STAT5 in specific cell types. HCQ use and the mean disease activity index (SLEDAI)-2K of each group are indicated at the bottom. SLE, systemic lupus erythematosus;HCQ, hydroxychloroquine.

The basal STAT signaling transduction varied significantly within one SLE patient, and between the SLE patient samples. The activation of STAT3 was increased in SLE T cells and monocytes, as was the activation of STAT5 in SLE T cells and B cells, relative to that in HDs. STAT signaling increased in some patients but decreased or disappeared in others. For example, SLE patient 10 showed STAT5 activation in his B cells and T cells, but lower pSTAT3 was detected in his T cells and monocytes. In SLE patient 01, STAT5 was activated in his T cells, but the phosphorylation of STAT5 was lower in his B cells, and pSTAT3 was reduced in his T cells and monocytes (Figure 3).

The SLE patients were further clustered with the HCE3.5 package (Hierarchical Cluster Explorer), according to the basal activation of the STAT3 in their mononuclear cells and T cells, and of STAT5 in their B cells and T cells. Each grid represents the phosphorylation of a STAT protein in one cell type. The phosphorylation of each STAT protein was scaled relative to the minimum phosphorylation level among the 20 SLE samples. Unsupervised clustering identified two main groups of SLE patients (Figure 3). The patients of group 1 were primarily defined by potentiated STAT5 signaling in their T cells and B cells, whereas there was no detectable activation of STAT5 in the B cells of the patients in group 2.

To investigate whether the phospho-profiling of STAT proteins might be related to lupus activity, we compared the clinical manifestations of the two groups identified with hierarchical clustering (Table 2). SLEDAI-2K and the erythrocyte sedimentation rate (ESR) were higher in the patients of group 2 (9.444±1.98 and 45.43±8.96 mm/h, respectively) than those in group 1 (4.364±0.9271 and 16±3.613 mm/h, respectively) (P = 0.0237 and P = 0.0052, respectively). The patients in group 2 had lower C3 levels than those of group 1, but this difference was not significant.

**Table 2:** Comparison of SLE patients in the two clusters.

Variable	Group 1 (n = 11)	Group 2 (n = 9)	P Value
Disease duration, years	4.425±1.543	6.412±2.827	NS
Rash	6 (54.54%)	5 (55.55%)	NS
Arthritis	0	0	NS
Vasculitis	2 (18.18%)	1 (11.11%)	NS
Oral ulcers	0	0	NS
Proteinuria	2 (18.18%)	2 (22.22%)	NS
SLEDAI-2K†	4.364±0.9271	9.444±1.98	0.0237
ESR, mm/h†	16±3.613	45.43±8.96	0.0052
C3, mg/dL	0.7609±0.06341	0.6256±0.05771	NS
Low complement	4 (36.36%)	7 (77.78%)	NS
WBC	0	1 (11.11%)	NS
Hgb	2 (18.18%)	0	NS
Autoantibody profile			
Anti-Ro	NS	2 (22.22%)	NS
Anti-U1 RNP	0.0281	2 (22.22%)	NS
Anti-Sm	4 (36.36%)	2 (22.22%)	NS
Anti-dsDNA	4 (36.36%)	6 (66.67%)	NS
No. autoantibodies¥	1.364±0.0643	1.556±0.4120	NS
Medical therapy			
Immunosuppressants	8 (72.72%)	2 (22.22%)	NS
Prednisone dose, mg/day§	17.50±3.873	17.2±5.535	NS
HCQ	7 (63.64%)	1 (11.11%)	0.0281

Values presented are means ± SD or numbers (%) of patients, depending on whether the data are continuous or dichotomous. ESR = erythrocyte sedimentation rate; Hgb = hemoglobin; dsDNA = double-stranded DNA; HCQ = hydroxychloroquine; NS = not significant.

†Mann–Whitney test was used, instead of a t test, because the data were not normally distributed.

¥Autoantibodies included anti-dsDNA, anti-Ro, anti-La, anti-U1 RNP, and anti-Sm antibodies, with a range of 0–5.

§All glucocorticoid doses were converted to equivalent daily doses of prednisone.

No significant differences was found in either the clinical manifestations, such as rash, arthritis, vasculitis, oral ulcers, proteinuria, white blood cells, and hemoglobin, or the autoantibody profiles, including anti-Ro, anti-U1 RNP, anti-Sm, and anti-dsDNA, of the two groups. There was no statistically significant difference in the prednisone doses of the two groups. Hydroxychloroquine (HCQ) was used more commonly by group 1 patients than by group 2 patients (P = 0.0281; Figure 3, Table 2).

**Figure 4:**
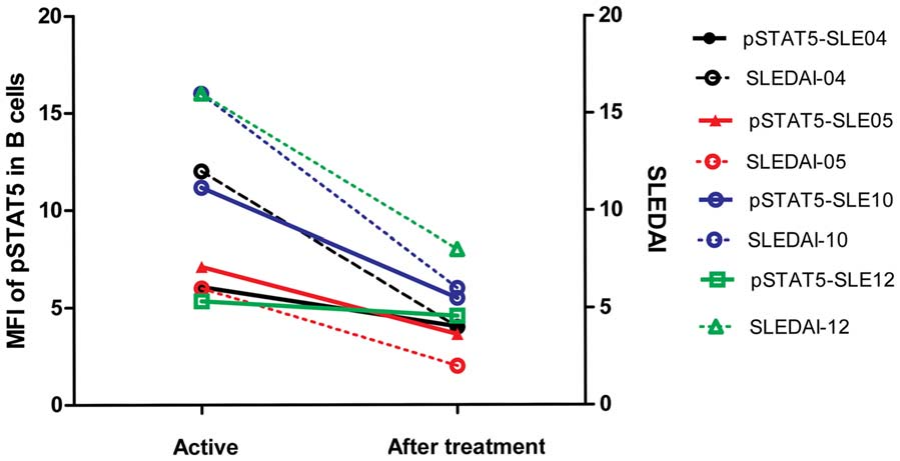
Phosphorylation of STAT5 was observed in patients at the beginning and after treatment. The expression of phosphorylated STAT5 in B cells was notably reduced in patients who had achieved significant clinical improvement after treatment. SLE-04, SLE-05, SLE-10, and SLE-12 represent four different patients.

To determine whether the activation of basal STAT signaling varies over time and parallels SLE activity, the cell-specific phosphorylation of STATs in four SLE patients with active lupus were re-analyzed after clinical remission. There was a positive correlation between STAT5 activity and lupus activity as assessed by SLEDAI-2K (Figure 4).

### STAT5 signaling in B cells is associated with inflammatory cytokines

**Figure 5:**
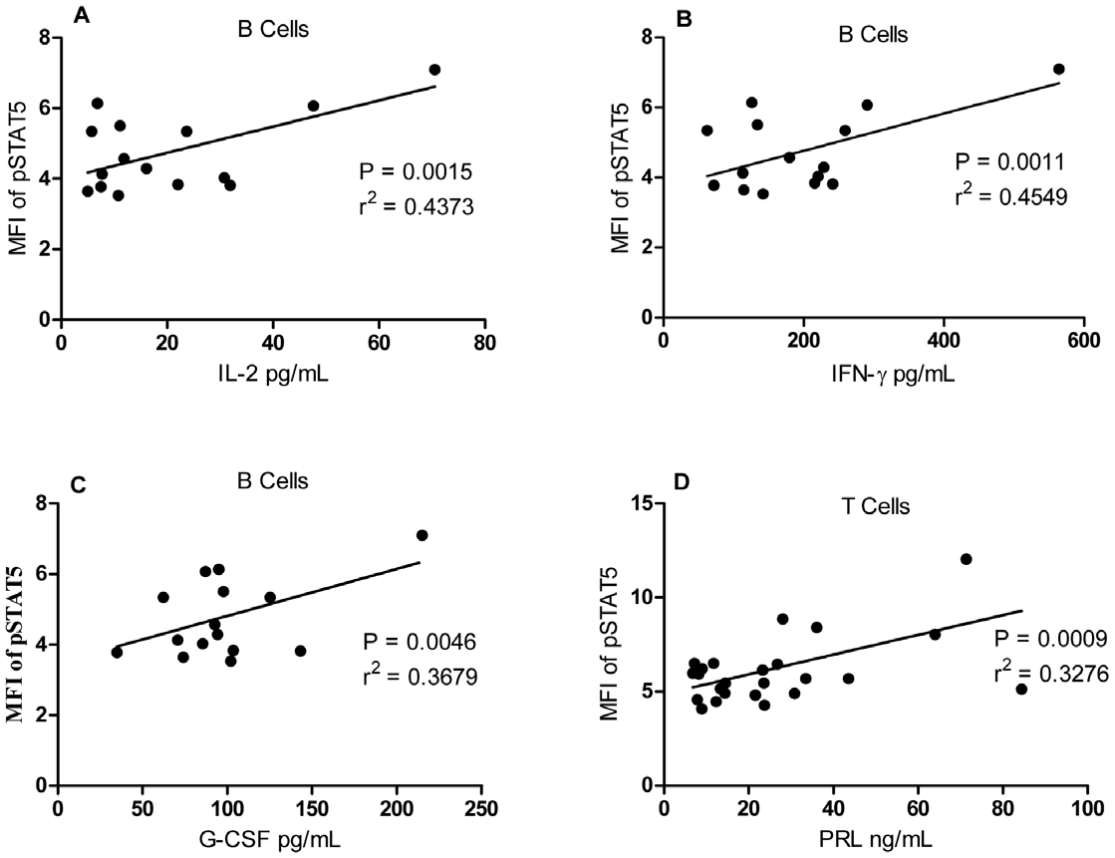
Association between the mean fluorescence index (MFI) of pSTAT5 in B cells and cytokine levels in SLE patients. The concentrations of serum IL2 (A), IFNγ (B) and G-CSF (C),correlated positively with the MFI of STAT5 in the B cells of SLE patients. (D) The serum prolactin concentration correlated positively with the MFI of pSTAT5 in the T cells of SLE patients. MFI, mean fluorescence index; IL, interleukin; IFNγ, interferon γ; G-CSF, granulocyte colony-stimulating factor; PRL, prolactin.

STAT5 activation in B cells correlated with the activity of SLE, as estimated as SLEDAI-2K. We then asked whether the inflammatory cytokines influence STAT5 signaling in lupus patients. A panel of 27 cytokines was detected in the sera of SLE patients using Bio-Plex™ Cytokine Assays. A series of cytokines, including IL2 (P = 0.0015, r^2^ = 0.44 ; Figure 5A), IFNγ (P = 0.0011, r^2^ = 0.45; Figure 5B), and G-CSF (P = 0.0046, r^2^ = 0.37; Figure 5C) were all correlated positively with STAT5 phosphorylation in B cells.

### Phosphorylation of Stat5 in T cells is associated with serum prolactin

PRL–STAT5 signaling was observed in the pathogenesis of cancer, including breast cancer [22]–[25]. In this study, we found high STAT5 activity in the T cells and B cells of SLE patients. To identify whether serum PRL levels are associated with the activation of STAT5 signaling in T and B cells, we compared the percentage of T cells and B cells with STAT5 activity and serum PRL levels. The percentage of T cells with phosphorylated STAT5 correlated positively with serum PRL (r^2^ = 0.33, P = 0.0009; Figure 5D).

### Reduced STAT signaling in response to IFNα and IL6 cytokines

Most cytokines activate STAT proteins that are phosphorylated at certain residues when they combined with specific surface receptors on the immune cells. We found basal levels of STAT3 and STAT5 signaling activation in SLE patients. Quantifying the cell-type-specific STAT signaling responses to cytokines should help us to better understand the pathology of SLE. PBMC were stimulated ex vivo with the inflammatory cytokines IFNα, IFNγ, IL2, IL6, and IL10, which are reported to play a role in the pathogenesis of SLE. Cytokine-induced fold changes in STAT phosphorylation were compared with those of HDs.

**Figure 6:**
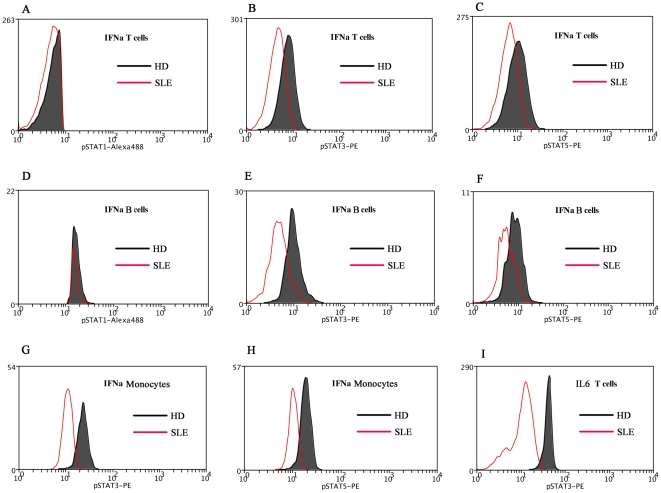
Comparison pSTATs expression between SLE patient and healthy donor (HD) after cytokine stimulation. A representative experiment is shown. The MFI for the phosphorylation of STAT1 (5A), STAT3 (5B), and STAT5 (5C) in the T cells of SLE patient was compared with that of HD. The MFI for the phosphorylation of STAT1 (5D), STAT3 (5E), and STAT5 (5F) in the B cells of SLE patient was compared with that of HD. The MFI for the phosphorylation of STAT3 (5G) and STAT5 (5H) in the monocytes of SLE was compared with that of HD. The MFI for pSTAT3 (5I) in the T cells of SLE patient was compared with that of HD. MFI, mean fluorescence index; SLE, systemic lupus erythematosus; HD, healthy donor; IFNα, interferon α.

**Figure 7:**
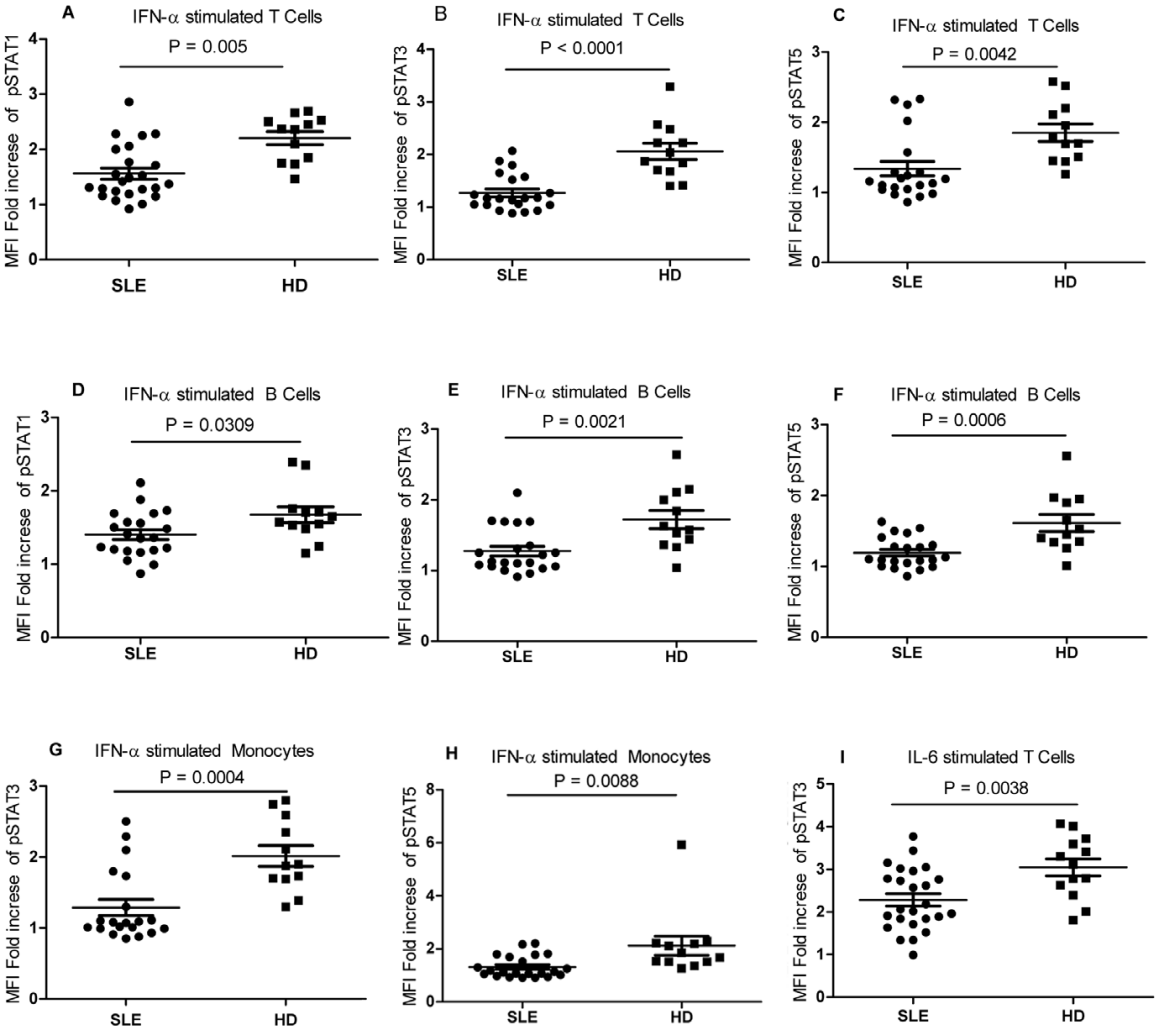
pSTATs in SLE patients after cytokine stimulation. The MFI for the phosphorylation of STAT1 (5A), STAT3 (5B), and STAT5 (5C) in the T cells of SLE patients decreased significantly after IFNα stimulation for 15 min. The MFI for the phosphorylation of STAT1 (5D), STAT3 (5E), and STAT5 (5F) decreased in the B cells of SLE patients after IFNα stimulation for 15 min. The MFI for the phosphorylation of STAT1 (5G) and STAT3 (5H) in the monocytes of SLE patients decreased after IFNα stimulation for 15 min. The MFI for pSTAT3 (5I) decreased significantly in the T cells of SLE patients after IL6 stimulation for 15 min. MFI, mean fluorescence index; SLE, systemic lupus erythematosus; HD, healthy donors; IFNα, interferon α.

In general, there was a reduced signaling response to certain cytokines in SLE patients compared with those in HDs. The responses of STAT1, STAT3, and STAT5 to IFNα were greatly reduced in SLE T cells, B cells, and monocytes, except for the activation of STAT1 in monocytes. The STAT3 response to IL6 was reduced in SLE T cells. Although there was enhanced responsiveness to IL10, but this difference in SLE patients was not statistically significant (Figure 6, Figure 7).

## Discussion

Cytokine network dysregulation is highly complex in SLE, Here, we focused on JAK–STAT signaling,which is one of the main intracellular signaling pathways activated by cytokines. The activation of STAT3 was observed in the monocytes and T cells of SLE patients, which was consistent with previous findings that SLE T cells exhibited higher levels of STAT3 and phosphorylated STAT3 [26]. Karonitsch T et.al found that STAT-1 expression was increased in PBMCs from SLE patients, SLE monocytes showed a considerably higher increase in pSTAT-1 expression upon IFNγ stimulation than monocytes from healthy individuals [9]. But we did not find the same result. One of the reason may be caused by heterogeneity of the SLE patients such as the genetic background of SLE patients, for all of our patients were Chinese. We also need increase our sample number to further confirm this phenomenon.

STAT5 signaling is also activated in lupus patients. In this study, STAT5 phosphorylation was much higher in group 2 SLE patients than in group 1 patients, which were segregated by clustering analysis, and the patients in group 2 were also confirmed as having more disease activity evaluated using the SLEDAI-2K and ESR than group 1 (Figure 2). The MFI of phosphorylation of STAT5 in B cells of PBMC significantly reduced when disease going into remission, which indicated that the STAT5 signaling in B cells also correlated with the disease status (Figure 3).

STAT5 is known to be activated by a wide range of cytokines, including the common γ-chain (γc) family, the IL3 family, single-chain receptors, class ΙΙ receptors, and growth factors [27]. We then asked whether these cytokines influence STAT5 signaling in SLE. A cytokine panel was detected in the sera of SLE patients, and we found that cytokines of the common γ-chain (γc) family (including IL2), the IL3 family (including G-CSF), and class ΙΙ receptors (including IFNγ) all correlated with STAT5 phosphorylation in B cells (Figure 5). All these cytokines were elevated in SLE patients and most of them were more highly elevated in the patients with active disease (data not shown).

There was no correlation between the phosphorylation of STAT5 in T cells and lupus disease activity or cytokine secretion. Hyperprolactinemia has been confirmed in SLE patients (20%–30%), and those with active SLE display higher serum PRL levels. PRL activates STAT5 when it binds to the prolactin receptor and induces immunoglobulin synthesis and anti-dsDNA in SLE lymphocytes [28]–[30]. Our study indicates that PRL–STAT5 signaling is cell-type specific, and only influences T cells, but not B cells or not monocytes.

The STAT5 signaling in T cell and B cells may provided as new therapy target in future such as inhibition the phosphorylation of STAT5, its cytokine receptors or other molecules of STAT5 signal pathway. Decrease the prolactin in the serum will be another treatment target in SLE. Bromocriptine, a dopamine analog, has been suggested be beneficial in protecting the SLE patients from disease relapse and in reducing the usage of steroid and immunosuppressant by suppressing the circulating prolactin [31]–[33]. Our results showed the therapeutic mechanism of the bromocriptine in SLE and will help us to expand the use of bromocriptine in SLE.

Strong negative regulation of the signaling response to the cytokines IFNα and IL6 was also observed, which has a causal role in human SLE. This result is consistent with the observation of Hale et al. in an lpr mouse model of lupus [16].

Hydroxychloroquine were used more frequently by the patients in group 1 than by those in group 2. Chloroquine, an antimalarial agent, is a drug commonly used in the treatment of SLE. A previous study showed that chloroquine can inhibit lymphocyte proliferation. Our results show that hydroxychloroquine might exert its antiproliferative effect by inhibiting JAK–STAT signaling.

In summary, basal activation of STAT signaling and a reduced response to the cytokines IFNα and IL6 were observed in the peripheral blood of SLE patients. The phosphorylation of STAT5 is associated with cytokines such as PRL, IL2, G-CSF, and IFNγ, which exert their biological effects through cytokine receptors and cell-type specifically. STAT5 signaling is very important in the regulation of the immune response in SLE. The basal activation of STATs signaling and reduced response to cytokines may be helpful us to identify the activity and severity of SLE although larger sample numbers still need to further confirm our result.
